# Protocol for generation of a 3D humanized lung-tumor-on-a-chip model of immune-hot and immune-cold microenvironments

**DOI:** 10.1016/j.xpro.2026.104732

**Published:** 2026-07-23

**Authors:** Sabrina Brito Añez, Sanne van Kesteren, Peter Carmeliet, Anna Rita Cantelmo, Patricia Yagüe, Jeffrey Kroon

**Affiliations:** 1 Laboratory of Angiogenesis and Vascular Metabolism, Department of Oncology and Leuven Cancer Institute (LKI), KU Leuven, VIB Center for Cancer Biology, VIB, 3000 Leuven, Belgium; 2 Amsterdam Cardiovascular Sciences, Atherosclerosis & Aortic Disease, Amsterdam, the Netherlands; 3 Amsterdam UMC Location University of Amsterdam, Department of Experimental Vascular Medicine, Meibergdreef 9, Amsterdam, the Netherlands; 4 Center for Biotechnology, Khalifa University of Science and Technology, Abu Dhabi, United Arab Emirates; 5 Université de Lille, Inserm, CHU Lille, Institute Pasteur de Lille, U1011-EGID, 59000 Lille, France

**Keywords:** Cell culture, Flow cytometry, Cancer, Immunology

## Abstract

The tumor microenvironment (TME) modulates immune responses and clinical outcomes. Here, we present a protocol to establish a fully humanized immune-hot and -cold lung TME using the Emulate organ-on-a-chip system by culturing human lung microvascular endothelial cells and human lung tumor cells. We describe steps for perfusing human peripheral blood mononuclear cells (PBMCs), visualizing immune cell recruitment, and analyzing immune infiltration across TME phenotypes through immunofluorescence, flow cytometry, or secretome analysis. This protocol can be adapted to model the TME in different cancer types.

## Highlights

• Steps to establish a fully humanized lung tumor-on-a-chip microenvironment
• Guidance for immunofluorescence staining and imaging in organ-on-a-chip devices
• Flow cytometry-based quantification and characterization of infiltrating immune cells
• Multiplex cytokine analysis to evaluate tumor microenvironment signaling

## Abstract (editor highlights)

Publisher’s note: Undertaking any experimental protocol requires adherence to local institutional guidelines for laboratory safety and ethics.

## Graphical abstract

**Figure:**
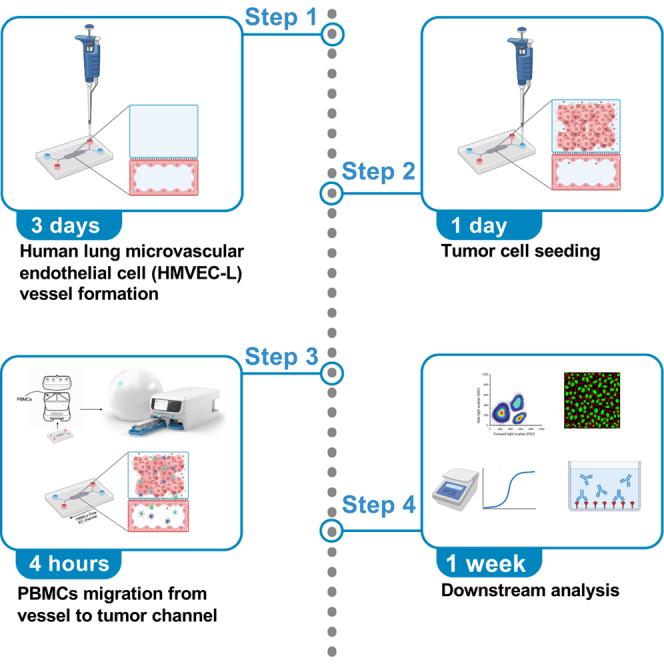

## Before you begin

The tumor microenvironment (TME) is a dynamic and complex ecosystem that critically influences cancer progression and therapeutic response. Depending on immune cell infiltration and inflammatory status, tumors can be classified as immune-hot or immune-cold, with the latter generally showing greater resistances to immunotherapies.^1^^,^^2^ Within the TME, endothelial cells (ECs) are key regulators of immune cell trafficking and intracellular communication.^3^ In tumor, ECs frequently adopt a pro-tumorigenic and immunosuppressive phenotype, thereby actively contributing to tumor immune evasion.^3^

Understanding the interactions among tumor cells, ECs, and immune cells within the TME is essential for developing effective cancer therapies. However, studying these interactions requires models that can recapitulate the physical and spatial complexity of human tissues. Traditional static 2D cultures do not recapitulate physiological flow and shear stress,^4^ while animal models often lack human-specific immunological features and limit a real-time visualization of cellular interactions at the vascular interface.^5^ To address these limitations, we developed a fully humanized tumor-on-a-chip platform.^6^ This protocol describes the steps required to establish a fully humanized lung TME using the Emulate microfluidic organ-on-a-chip system. In this model, we utilize H2009 and A549 lung adenocarcinoma cell lines are used, each harboring distinct co-mutations associated with differential immunosuppressive phenotypes. Notably, A549 cells exhibit a more immunosuppressive profile than H2009 cells and are cultured in the upper channel, representing the tumor compartment. In parallel, human lung microvascular ECs (HMVECs-L) are seeded in the lower vascular channel to mimic the lung microvasculature. Freshly isolated human peripheral blood mononuclear cells (PBMCs) are perfused through the vascular channel to investigate immunomodulatory interactions at the immune cell-endothelium interface and to characterize immune cell trafficking toward the tumor compartment.

This dual-channel configuration enables dynamic co-culture under continuous perfusion, accurately recapitulating *in vivo* blood flow and interstitial fluid dynamics, thereby promoting physiologically relevant cell-cell interactions. As such, the platform provides a robust tool to elucidate the complex mechanisms by which ECs contribute to tumor progression and the establishment of an immunosuppressive TME in lung cancer. Although optimized for lung cancer, the protocol can be adapted to other cancer cell types or patient-derived primary tumor cells following appropriate optimization of culture conditions and co-culture parameters.

### Innovation

Our lung tumor-on-chip protocol represents a significant advancement over conventional *in vitro* preclinical models (e.g., 2D and basic 3D cultures) which often fail to recapitulate the complex biochemical and biophysical factors and critical immunomodulatory interactions of the *in vivo* TME.^6^ These models are typically avascular and static, resulting in an unrealistic immune cell migration that occurs passively rather than under physiological flow. Consequently, they fail to capture the critical endothelial and immune cell interactions essential for modeling hot and cold TMEs.^4^ Moreover, our model provides a fully humanized representation of the TME, unlike other *in vivo* models, such as xenograft models in mice. Although they provide systemic complexity, species-specific differences in immune cell signaling and trafficking limit their translational potential.

Unlike many custom-built or fragmented organ-on-chip setups, which are often not standardized and thus introduce variability in downstream readouts, Emulate system provides standardized Organ-Chips and automated instrumentation, reducing experimental variability and simplifies complex workflows. Our vascularized tumor-on-chip design enables robust cell-cell interactions and paracrine signaling between distinct cell populations (e.g., tumor cells and ECs) through a porous membrane separating the channels, while maintaining independent perfusion in the vascular channel.

This protocol uniquely enables the investigation of dynamic endothelium-immune cell-tumor cell interactions under continuous flow, thereby providing mechanistic insights into cancer progression and therapeutic resistance mechanisms.

### Institutional permissions

All blood donors provided informed consent for their participation in this study, in accordance with the Amsterdam UMC Blood Sampling for Biomedical experiments (BACON) protocol. The Medical Ethics Committee of the Amsterdam UMC determined that this specific study protocol did not fall under the Medical Research Involving Human Subjects Act, and therefore formal ethics committee approval was not required d. However, it is crucial for users to obtain informed consent from participants and ensure full compliance with their institutional guidelines for the use materials derived from healthy volunteers.

### Culturing HMVECs-L and A549 and H2009 tumor cell lines

**Timing: ∼1 week**

1. Culture HMVECs-L in endothelial growth medium (EGM-2 Basal Medium; Lonza) by adding the complete bullet kit to endothelial base medium (EGM-2 MV Microvascular Endothelial Cell Growth Medium SingleQuots; Lonza) with 10% fetal bovine serum (FBS; Merck; Biochrom). Culture A549 and H2009 cells in RPMI 1640 (Thermo Fisher Scientific) supplemented with 10% FBS, 100 IU/mL penicillin and 100 μg/mL streptomycin (Thermo Fisher Scientific) at 37°C, 5% CO^2^.

***Note:*** HMVECs-L can be cultured until approximately passage 8 (P8), after which they gradually lose endothelial characteristics and proliferative capacity.

**CRITICAL:** HMVECs-L should always be seeded at approximately 40% confluence and passaged at ±80% confluence for optimal growth. HMVECs-L require cell-cell contact-derived signals to proliferate, and over confluency may lead to loss of their proliferative capacity. Additionally, HMVECs-L medium should be refreshed every other day to maintain optimal concentrations of required supplements.

2. Keep the HMVECs-L, A549 and H2009 cells in culture for at least two passages before seeding them in the chip.
3. Check the cell lines for mycoplasma on a regular basis.

### Chip activation

**Timing: ∼1 week**

https://emulatebio.com/The steps for preparing the chips are based on the Chip-S1 Basic Research Kit Protocol. The complete and detailed instructions can be found on the Emulate website (https://emulatebio.com/).

***Note:*** Chip activation solution should be prepared right before use and protected from light, as it is light sensitive. 200 μL of ER-1 solution will fill approximately three S1-chips. Once activated, chips can be stored for up to 2 weeks at 4°C with sterile PBS in both channels and with PBS-filled filter tips inserted into all the inlet and outlet ports.

**CRITICAL:** Bubbles should be avoided during the whole activation process as they may impair chip functionality (coating, cell adhesion and flow). If bubbles are present, the solution should be aspirated and re-pipetted to remove them.

## Key resources table

**Table:** 

REAGENT or RESOURCE	SOURCE	IDENTIFIER
**Antibodies**		

CD4-BV605 1:50	BD	BD566908
CD8-BV650 1:50	BD	BD565289
CD3-BV786 1:200	BD	BD563800
CD45-BB515 1:200	BD	BD564585
Viability-ef780 1:4000	Invitrogen	65-0865-14
CD31-APC 1:50	Biolegend	BL303116
CD19-RY703	BD	BD571429
CD56	BD	BD571400
HLA-DR-PE 1:100	BD	BD560943
Cell Tracker Green CMFDA 1:1000	Celltracker	C7025
Cell Tracker Deep red 1:1000	Celltracker	C34565
Ve-cadherin 1:100	R&D	AF 1002
Phalloidin Alexa Fluor 555 1:40	Cell Signaling Technology	8953
Mouse anti-human CD31 Alexa Fluor 647 1:200	BD Pharmingen	561654
DAPI 1:5000	BioLegend	422801
Hoechst 1:500	Thermo Fischer Scientific	62249

**Biological samples**		

Human whole blood	Amsterdam UMC, location AMC	N/A

**Chemicals, peptides, and recombinant proteins**		

RPMI 1640	Gibco	11875093
EBM-2 Basal Medium	Lonza	CC-3156
EGM-2 MV Microvascular Endothelial Cell Growth Medium SingleQuots	Lonza	CC-4147
Fibronectin	Corning	354008
Collagen type IV	Sigma	C6745
Accutase	Merck	A6964
PBS	Gibco	10010–015
Formaldehyde solution	Sigma-Aldrich	F8775
Bovine serum albumin	Sigma-Aldrich	A3912
Triton X-100	Sigma-Aldrich	T8787
Trizol	Invitrogen	15596018
UltraPure 0.5M EDTA	Invitrogen	15575020
Fetal Bovine Serum	Gibco	10270–106
Percoll	Cytiva	17089101
Gelzan	Sigma	G1910
Lymphoprep	STEMCELL Technologies	07851
Matrigel Matrix	Corning	354263
Cell recovery solution	Corning	354253

**Critical commercial assays**		

Chip-S1 Basic Research Kit (including ER-1, ER-2, S1 chips and Pods)	Emulate	BRK-S1-WER-12

**Experimental models: cell lines**		

Human lung microvascular endothelial cells (HMVECs-L)	Lonza	CC-2527
A549	ATCC	CCL-185 ™
H2009	ATCC	CRL-5911 ™

**Software and algorithms**		

Las-X	Leica	https://www.leica-microsystems.com/products/microscope-software/p/leica-las-x-ls/
BD FACSDiva	BD Biosciences	https://www.bio-rad.com/en-nl/product/cfx-maestro-software-for-cfx-real-time-pcr-instruments?ID=OKZP7E15
Fiji	ImageJ	https://imagej.net/software/fiji/
Imaris v.10.1.1	Oxford Instruments	https://imaris.oxinst.com/
Biorender	BioRender	https://www.biorender.com/

**Other**		

Zoë-CM1 Culture Module	Emulate	N/A
Orb-HM1 Hub Module	Emulate	N/A
Steriflip- HV filters	EMD Millipore	SE1M003M00
UV light Box	Emulate	N/A
Chip cradle	Emulate	N/A
4 mm diameter discs of 50 μM thick PET	Emulate	N/A
Revolve microscope	Echo	N/A
Thunder microscope	Leica	N/A
BDFacsCantoII	BD	
BDSymphony	BD	
Adhesive PCR Plate Foils	Thermo Scientific	AB0626
Nunc 96-Well Polystyrene Conical Bottom MicroWell Plates	Thermo Scientific	243656
Leucocep tubes	Greiner Bio-one	227290
10 mL EDTA Vacutainer blood collection tubes	BD	367525

## Materials and equipment

### Equipment

Required equipment is listed in the key resources table, section other, above.

### Materials

Required materials are listed in the key resources table, section other, above. In addition, the preparation of the following media and solutions is needed during the protocol.

**Table:** EGM-2 MV media

Reagent	Final concentration	Amount
EBM™-2 Basal Medium	N/A	500 mL
Fetal Bovine serum	10%	50 mL
Hydrocortisone	N/A	0.20 mL
hFGF-B	N/A	2 mL
VEGF	N/A	0.5 mL
R3-IGF-1	N/A	0.5 mL
Ascorbic Acid	N/A	0.5 mL
hEGF	N/A	0.5 mL
GA-1000	N/A	0.5 mL
**Total**	**N/A**	**554.7 mL**

Complete EGM-2 MV can be stored for up to 3 months at 4°C.

**Table:** RPMI media

Reagent	Final concentration	Amount
RPMI 1640	N/A	500 mL
Fetal Bovine serum	10%	50 mL
Penicillin/streptomycin	1%	5 mL
**Total**	**N/A**	**555 mL**

Complete RPMI can be stored for up to 3 months at 4°C.

**Table:** RBC lysis buffer

Reagent	Final concentration	Amount
NaHCO_3_	10 mM	N/A
NH_4_Cl	160 mM	N/A
EDTA	1.3 mM	N/A
milliQ water	N/A	N/A

RBC lysis buffer can be stored for 1 year at 4°C.

**Table:** Buoyancy media

Buoyancy media		
Reagent	Final concentration	Amount
Percoll	N/A	2 mL
Gelzan	N/A	32 μL
**Total**	**N/A**	**2.032 mL**

The buoyancy medium should be freshly prepared immediately before use.

**Table:** Blocking solution for chip section staining

Reagent	Final concentration	Amount
Donkey serum	10%	N/A
Triton	0.5%	N/A
BSA	2%	N/A
PBS	N/A	N/A

The blocking solution medium should be freshly prepared immediately before use.

**Table:** MACS buffer

Reagent	Final concentration	Amount
BSA	0.5%	N/A
EDTA	2 mM	N/A
PBS	N/A	N/A

MACS buffer can be stored for 2 weeks at 4°C.

## Step-by-step method details

### Endothelial cell channel coating and seeding

**Timing: 2 days**

**Timing: ±5 h (for step 1)**

**Timing: ±1 h (for step 2)**

**Timing: 6 h (for step 3)**

**Timing: ±12 h (for step 4)**

**Figure 1:**
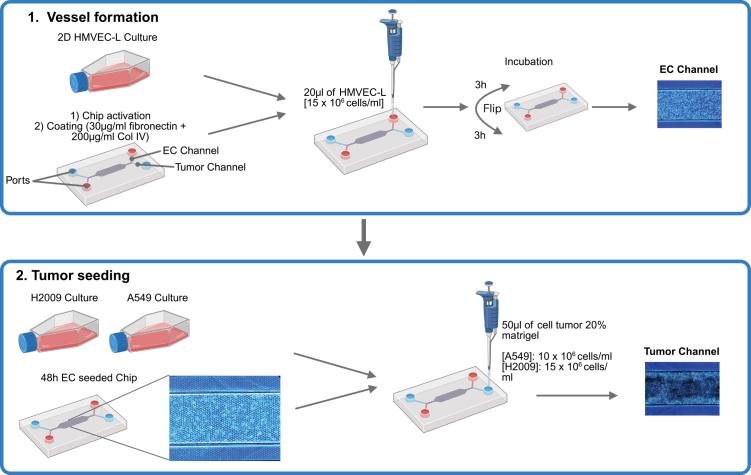
Cell seeding setup in chip channels

The following section describes the steps to fully form the endothelial vessel within the bottom channel of the chips. Figure 1 shows an overview of the cell seeding process in the chip channels.

1. Chip coating.
  The following step describes how to prepare the ECM solution and coat the chips.
  a. Thaw the fibronectin and collagen IV on ice.
  b. Prepare the appropriate volume for the required number of chips. Approximately, 100 μL should be prepared per chip at a concentration of 30 μg/mL for fibronectin and 200 μg/mL for collagen IV.
  c. When chips have been activated days in advance, fully aspirate the PBS that was introduced to store them.
  d. Carefully introduce the ECM solution through the bottom channel inlet until a small drop of ECM is formed in the bottom channel outlet.
  e. Repeat Step d for the top channel.
  f. Add a drop of ECM in the inlet ports to avoid any bubbles.
  g. Place the chips in a Petri dish to keep the chips sterile while inspecting the channels under the microscope to ensure that there are no bubbles present.
    ***Note:*** If bubbles are present, wash the channel with more ECM until all bubbles have been removed.
  h. Place the chips in a 150 mm culture dish and leave them at 4°C for 2 h.
    **Pause point:** Coated chips can be stored at 4°C for up to a week in sterile containers. For storage, add 1 mL sterile PBS to a 15 mL Falcon conical tube cap to provide extra humidity and avoid the chips drying out.
  i. Place the chips in an incubator (37°C, 5% CO_2_) for 2 h prior to cell seeding.
2. Cell seeding.
  When HMVECs-L reach 80% confluency after at least 2 passages in a T-75 flask, prepare them for seeding following the steps bellow.
  a. Wash the cells 1× with PBS.
  b. Detach the cells with accutase (1.5 mL for a cell culture flask T-75).
  c. Add 8.5 mL EGM-2 MV (full), resuspend and transfer to a 50 mL conical tube.
  d. Prepare an Eppendorf tube for cell counting by adding 10 μL trypan-blue.
  e. Pipette cells gently to create a homogeneous mixture and transfer 10 μL to the prepared Eppendorf tube.
  f. Count the cells with a counting chamber.
  g. Transfer the required volume of the cell suspension (3 × 10^5^ cells per chip) into a new 50 mL conical tube.
  h. Spin them down 5 min at 300 rcf.
    ***Note:*** The cell pellet will be very small. Aspirate carefully.
  i. Carefully aspirate the supernatant.
  j. Add 20 μL of EGM-2 MV (full) per chip (final concentration HMVECs-L is 15 × 10^6^ per mL) and gently resuspend the cells.
    **CRITICAL:** It is necessary to pipette the cells carefully and smoothly to ensure uniform chip seeding. Additionally, bubbles should be avoided; if bubbles occur, cells need to be reseeded.
    ***Note:*** Gently pipette up and down the cell suspension every time before seeding the cells into the chip to ensure a homogeneous seeding.
  k. Remove the ECM and wash both channels and wash both channels with 100 μL warm EGM-2 MV (full).
  l. Seed 20 μL of the HMVECs-L suspension (300.000 cells) into the bottom channel of one chip.
    **CRITICAL:** While seeding, remove the outflow with a tissue without touching the outlet port to avoid the aspiration of the HMVECs-L.
  m. Flip the chip upside down quickly once and back to the normal position.
    **CRITICAL:** Be careful that the HMVECs-L droplet will not move to the top inlet or outlets as this will result in HMVECs-L attaching in the top channel.
  n. Place the chips in a 150 mm Petri dish, cover the dish and transfer to the microscope to check the seeding density within the chip.
    ***Note:*** If seeding density is not optimal or uniform, or there are bubbles present, reseed the cells as quickly as possible.
3. Upside-down cell attachment.
  The following step describes how the chips are handled to allow the attachment of the cells in the upper part of the channel.
  a. Place the chips upside down in the Emulate chip cradle and keep the container humid with 1 mL sterile PBS in 15 mL falcon caps.
  b. Transfer the Petri dish with the chips to the incubator (37°C, 5% CO_2_) for approximately 3h, or until cells in the bottom channel have started to be attached.
    ***Note:*** Confirm attachment of cell by microscope.
  c. Once ECs have attached, flip the chips back to an upright position.
  d. After 3h, perform a gravity wash on the chips:
    i. Insert a filter tip with 200 μL of EGM-2 MV (full) in the bottom channel inlet.
      ***Note:*** This should cause the medium to gently flow through the channel.
    ii. Place an empty 200 μL filter tip on the bottom channel outlet which will collect the excess medium.
      **CRITICAL:** If there is no medium flow during the gravity wash, flow should be ensured by slightly moving the tip in case there is any bubble impeding the flow or, by smoothly pipetting a small amount until the bubble is ejected from the channel and the flow is observed.
4. Cell attachment.
  a. Repeat step h in the top channel to ensure nutrient availability for the bottom channel cells.
  b. Place filter tips filled with medium on all inlets and outlets to keep the channels sterile and supply the cells for approximately 1 day with nutrients.
    ***Note:*** Chips can now be kept outside sterile containers.
  c. Verify cell attachment and morphology under a phase-contrast microscope (Figure S1A).
  d. Incubate chips from 12 to 16 h in the incubator (37°C, 5% CO_2_).
  e. Repeat the gravity wash according to step 3h-i.
  f. Check the cells seeding under the microscope (Figure S1A) to assess their growth.
  g. Incubate chips from 12 to 16 h in the incubator (37°C, 5% CO_2_).

#### Tumor cell seeding

**Timing: ±2 h**

The following section describes the required steps for the preparation and seeding of the tumor cells in the chip.

5. When A549 and H2009 cells reach 80% confluency after at least 2 passages, prepare them for seeding.
  a. Wash the cells once with PBS.
  b. Detach the cells with trypsin (1.5 mL for a cell culture flask T-75).
  c. Add 8.5 mL RPMI (full), resuspend and transfer to a 50 mL conical tube.
  d. Prepare an Eppendorf tube for cell counting by adding 10 μL trypan blue.
  e. Pipette cells gently to create a homogeneous mixture and transfer 10 μL to the prepared Eppendorf tube.
  f. Count the cells with a counting chamber.
  g. Transfer the required volume of the cell suspension (500.000 cells/chip for the A549 tumor cells and 750.000 cells/chip for the H2009 tumor cells) in a new 50 mL conical tube.
    ***Note:*** Different seeding densities are used for the two tumor cell lines, due to their different cell size and volume they occupy in the channel.
  h. Spin them down 5 min at 300 rcf.
  i. Carefully aspirate the supernatant.
  j. Add 40 μL of RPMI (full) per chip (final concentration A549 is 12.5 × 10^6^/mL; H2009 is 18.75 × 10^6^/mL in medium), gently resuspend the cells and keep them on ice.
6. Seed tumor cells.
  ***Note:*** Tumor cells are seeded in Matrigel to mimic a 3D tumor and avoid a monolayer culture. Other replacements of matrix could be used if optimized for the used cell type.
  a. Thaw the Corning Matrigel Growth Factor Reduced (GFR) Basement Membrane Matrix, Phenol Red-free, LDEV-free.
    **CRITICAL:** As Matrigel polymerizes rapidly, it is necessary to keep it on ice during all the steps mentioned below. Additionally, it is recommended to also work with chilled tips and Eppendorf tubes to delay polymerization.
  b. Prepare the seeding solution by mixing 40 μL of tumor cells solution (prepared in step 4) with 10 μL of Matrigel (20% Matrigel). The final cell concentration per chip should be 10 × 10^6^/mL for A549 and 15 × 10^6^/mL for H2009).
    **CRITICAL:** Gently resuspend the mixture to avoid bubble formation.
    **CRITICAL:** Keep the seeding solution on ice to prevent Matrigel polymerization.
  c. Carefully aspirate medium from the outlet of the top channel of one chip.
  d. Gently agitate cell suspension before seeding each chip to ensure a homogeneous cell suspension.
  e. Place an empty 200 μL non-filtered tip in the outlet of the top channel.
  f. Seed 50 μL of the tumor cell suspension into the top channel.
  g. Ensure the correct and homogenous seeding of the tumor cells by looking at the chips in the microscope (Figure 1).
    ***Note:*** If there are visible bubbles within the gel, quickly aspirate the mix and fill again. Tumor cells should appear as dense aggregates in the top channel (Figure S1B).
  h. Place the chip in the incubator (37°C, 5% CO2) for 2 h to allow the gel to polymerize.

#### Chip preparation for culture in the Zoë module

**Timing: 5 h 45 min total. Gas equilibration of media: 1 h 10 min. Prime pods: 5 min. Wash chips and PET disk placement: 10 min. Chip connection to pods: 5 min. Regulate cycle: 4 h 15 min**

This section is an abbreviated version of preparing the chip for culture in the Zoë module. Additional details can be found in the Chip-S1 Basic Research Kit protocol on the Emulate website (https://emulatebio.com/).

7. Gas equilibration media.
  ***Note:*** This is an essential step to reduce the bubbles in the media used during the culturing process and should not be avoided. The media used should always be kept warm left outside the incubator or water bath for no longer than 10 min.
  a. Place at least 3.3 mL of complete EGM-2 MV (full) medium for each chip in a 50 mL conical tube.
  b. Warm the media at 37°C for at least 1 h.
  c. Connect the tube containing the media to a Steriflip unit in the BSC. Attach the vacuum source to the Steriflip unit for 10 seconds.
  d. Invert the unit and apply vacuum for 5 to 10 min to remove the bubbles.
    ***Note:*** During the filtration in the Steriflip unit, the tube can be placed in a water bath to keep the temperature and avoid the formation of new bubbles.
  e. Detach the vacuum tubing from the Steriflip and detach the tubes in the BSC.
  f. Place the tube containing the media in the incubator (37°C, 5% CO_2_) with the cap loose until needed.
8. Prime pods.
  a. Label the pods accordingly to the corresponding chips.
  b. Place the required pods into the Zoë tray.
  c. Add 1 mL of EGM-2 MV (full) media to the pod outlet reservoir of the top channel (gel channel).
  d. Add 3 mL of pre-equilibrated media (step 6) to the inlet reservoir of the bottom channel (endothelial channel).
  e. Carefully pipette over the via of the outlet reservoir of the bottom channel PET (endothelial channel) 500 μL of pre-equilibrated media.
  f. Place the Zoë tray containing the pods into the Zoë module and run the Prime cycle twice.
  g. Confirm successful priming by examining the bottom of the pods; small droplets of media should be present at the ports. If not, re-run the Prime cycle.
  h. Wash chips and place a 4 mm diameter and 50 μm thick PET disk.
    ***Note:*** As the gel channel will not be perfused, the PET disk is used to cover the inlet port and avoid any pressure from the system that could damage the channel. Moreover, media is present in the outlet reservoir to keep the gel hydrated and nourished.
  i. Remove the tips from the chip inlet and outlet ports and place a drop of media on all of them.
    **CRITICAL:** If any air bubbles are observed in the perfusion channel (endothelial), they should be removed by performing a gravity wash (refer to step 3h). If tiny and limited air bubbles are present in the gel channel, they should not be removed as they may be cleared during the Regulate cycle.
  j. Place a PET disk onto the inlet of the gel channel with a pair of tweezers, taking care to center the disk over the ports.
  k. Using tweezers, carefully press the disk and remove the excess media from the droplet with a tissue.
    ***Note:*** Do not remove the other droplets from the ports, as this will help prevent bubbles when connecting the chips to the pods.
9. Connect the chips to the pods.
  a. Slide the chip carrier into the tracks on the bottom of the Pod until it is fully seated.
  b. Using your thumb, press the chip carrier tab gently until it clicks into the pod.
  c. Confirm that the PET disk is still placed on top of the center of the chip port.
  d. Remove any excess media on the surface of the chip with a tissue.
10. Place the pods into the tray and into the Zoë module.
11. Set up the culture conditions on the Zoë module for S1-chip.
  a. The flow rate for the gel channel should be set at 0 μL/h.
  b. The flow rate for the endothelial channel should be set at 10 μL/h without stretch and frequency set to 0.
12. Set the Regulate cycle in the Zoë module (approximately 2 h per cycle).
13. Check if the gel compartment is intact and medium collected in the bottom channel reservoir is similar between different pods.

**Pause point:** The second Regulate cycle can be run the day after.

14. Run a second Regulate cycle.

### PBMC migration

**Timing: 8 h 20 min total**

**Timing: 2 h (for step 15)**

**Timing: 2 h (for step 16)**

**Timing: 4 h 30 min (for step 17)**

**Figure 2:**
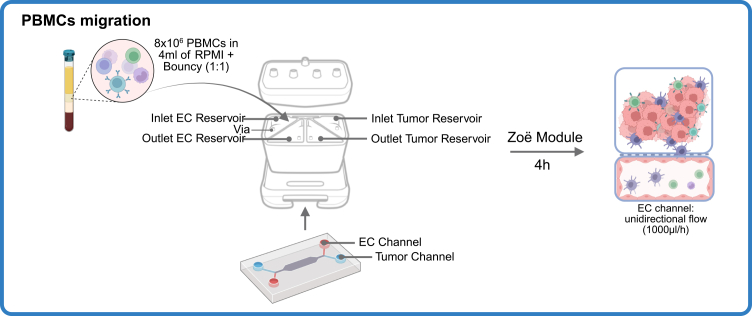
PBMC migration workflow

This section of the protocol describes the steps to isolate PBMCs, prepare the buoyancy media to perfuse the endothelial channel, and induce PBMC migration. Figure 2 provides a schematic representation of this process.

15. PBMCs isolation.
  ***Note:*** Here, PBMCs were isolated from whole blood obtained from healthy donors who have given informed consent. PBMCs can also be previously isolated and frozen for later use.
  a. Collect 2x 10 mL EDTA blood from a donor.
  b. Add 15 mL Lymphoprep to 2×50 mL Leucosep tubes.
  c. Spin down at 1000 rcf for 30 s at 21°C with maximum brake.
  d. Transfer the blood into 2×50 mL falcon tubes.
  e. Rinse empty blood tubes 2x with PBS-EDTA (±4 mL) and transfer any remaining blood into the falcon tubes.
  f. Dilute the blood with PBS-EDTA to an end volume of 35 mL.
  g. Add 35 mL diluted blood into 50 mL Leucosep tube (containing lymphoprep).
  h. Centrifuge at 1000 rcf for 15 min without brake (acceleration 9 – deceleration 1) at 21°C.
  i. Carefully remove ∼15 mL of the plasma layer using a 10 mL pipette.
  j. Decant the PBMCs layer into 50 mL falcon tubes, transferring the PBMCs from each tube into a separate new tube.
  k. Top up with cold PBS-EDTA.
    ***Note:*** From now onward work on ice.
  l. Centrifuge at 300 rcf for 10 min at 4°C to remove Lymphoprep, then aspirate the supernatant.
  m. Remove the supernatant and resuspend the pellet in 3 mL red blood cell (RBC) lysis buffer (10 mM NaHCO_3_, 160 mM NH_4_Cl, 1.3 mM EDTA, pH 7.4 in milliQ water) for 4 min on ice.
  n. Deactivate RBC lysis buffer by topping up with cold PBS-EDTA.
  o. Centrifuge at 300 rcf for 10 min at 4°C, then aspirate the supernatant. Repeat RBC lysis (step 15: m. - o.) if erythrocytes are still visible.
  p. Resuspend the cell pellets in RPMI (full) and pool in one 50 mL falcon tube.
  q. Count cells using your preferred counting method.
  r. Prepare a 2 mL solution of 8×10^6^ PBMCs per chip.
16. Preparation of buoyancy media.
  a. Warm 2 mL Percoll per chip at 37°C for at least 1 h.
  b. Add 32 μL Gelzan to every 2 mL of Percoll.
  c. Mix the buoyancy medium thoroughly, avoiding the formation of bubbles.
  d. Warm the buoyancy medium at 37°C for at least 30 min.
  e. Connect the tube containing the buoyancy medium to a Steriflip-unit without flipping the tube.
  f. Apply vacuum for 15 min without filtering.
17. PBMCs perfusion and migration.
  a. Mix 2 mL PBMC suspension with 2 mL of buoyancy media per chip (1:1).
  b. Keep at 37°C until needed for perfusion.
  c. Take the tray containing the chip from the Zoë module.
  d. Remove the media from the bottom inlet reservoir and bottom outlet reservoir (endothelial channel).
  e. Mix the PBMC solution gently.
  f. Add 4 mL of PBMC solution to the bottom inlet reservoir (endothelial channel) of each pod.
  g. Pipette over the via of the outlet reservoir of the bottom channel (endothelial channel) 500 μL of pre-equilibrated media.
  h. Place the chip into the Zoë module.
  i. Set flow rates to 1000 μL/h for the endothelial channel in the Zoë module.
    ***Note:*** After 15 min of flow, the chips can be checked under the microscope to confirm proper PBMC migration and flow.
  j. Let the PBMCs migrate for 4 h.

### End of chip culture in the Zoë module

**Timing: 15 min**

**Figure 3:**
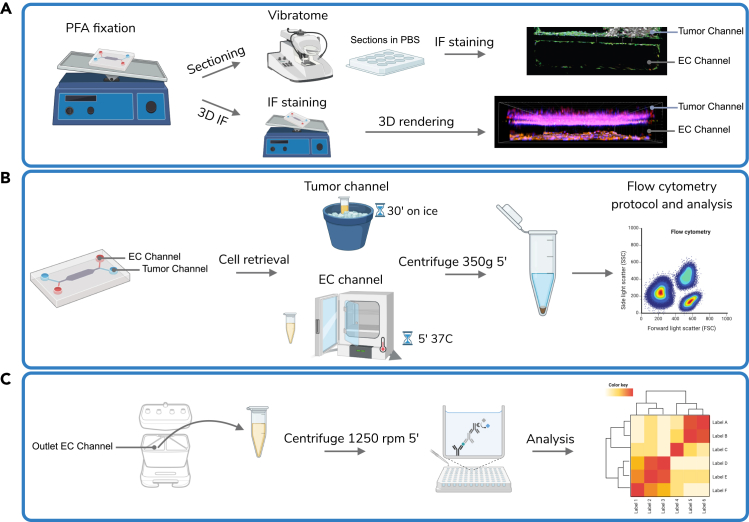
Optimized assay readouts (A) Chip fixation, sectioning and staining for immunofluorescence. (B) Cell retrieval and flow cytometry. (C) Secretome analysis.

This section describes the end of the culture in the Zoë module and references all the available optimized readouts for the S1 chips. Figure 3 shows a detailed overview of the optimized assays.

18. After PBMC migration, pause the Zoë module and remove the tray containing the chips.
19. Using a microscope, representative images of the chips can be taken if required.

***Note:*** The imaging distance from the bottom of the Chip-S1 to the top of the membrane is 850 μm; therefore, objectives with a working distance of at least 1 mm are required to resolve cells in both the bottom channel and on the membrane surface. If the full top channel also needs to be acquired, a longer-working-distance objective is required, as the top channel adds an additional 1000 μm imaging depth.

20. From the bottom outlet reservoir (endothelial channel) media can be collected for further analysis.

***Note:*** Stored collected samples at −80°C to prevent degradation of proteins and other soluble components.

21. Aspirate any media left that is not used for analysis, ensuring that a small amount of media is left to cover the reservoir vias so bubbles are not formed.
22. Choose which chips will be used for each analysis.
23. Remove chips from the pods and continue with the downstream processing.

***Note:*** Different analysis can be performed as readout of this experiment, such as immunofluorescence imaging of whole chips or chip sections, flow cytometry analysis of tumor and endothelial channels, qPCR on tumor or ECs and secretome analysis (ELISA or multiplex).

### Chip fixation, sectioning, and immunofluorescent staining

**Timing: 6.5 days total**

**Timing: 1 h 30 min (for step 24)**

**Timing: 1 day (for step 25)**

**Timing: 2 days (for step 26)**

**Timing: 2 days (for step 27)**

**Timing: 8 h (for step 28)**

This section describes the procedure to follow with the chips selected for imaging (Figure 3A).

24. Chip fixation.
  a. Remove the PET disk from the inlet of the gel channel.
  b. Carefully plug in both outlets of the chip and the inlet of the gel channel with 200 μL non-filtered tips (Figure S2).
  c. Using a 200 μL tip, take up 200 μL of 4% paraformaldehyde (PFA). Pipette slowly into the bottom channel, until about half of the PFA has been pipetted in.
  d. Leave the pipette plugged in the inlet to allow the PFA to flow through.
  e. Add 200 μL of 4% PFA carefully to the tip in the bottom channel inlet bringing the volume up to 400 μL.
  f. Incubate for at least 1 h at 20°C–25°C on a rocker, ensuring gentle flow of PFA through the channel.
    **CRITICAL:** The rocking should be very slow, as the cells are still undergoing fixation.
  g. Remove the tips from all the inlets and outlets and replace them with new 200 μL tips.
  h. Wash by placing a 200 μL tip with PBS to the bottom channel, by leaving it in the inlet the PBS will flow by gravity through the top channel.
  i. After a minimum of 15 min, remove the tips and repeat the PBS gravity wash 2x.
    **Pause point:** After the washes, the chips can be stored up to 2 weeks at 4°C. Ensure that while stored, the chip never dries out, as this could cause the formation of crystals from the PBS in the channels.
25. Chip sectioning.
  Methods Video S1. Step-by-step procedure for chip sectioning.
  a. Using a razor blade, cut as much as possible the poly-dimethylsiloxane membrane (PDMS) around the channels of the chip.
  b. Mount the chip channels using Pattex instant glue in the specimen tube.
  c. Once it is dry, using a semi-automated vibrating microtome, place the specimen tube in the holder.
  d. Cut 15 μm thick slices and keep in PBS until used for staining.
  Methods Video S1. Step-by-step procedure for chip sectioning, related to Step 25
26. Chips section staining.
  a. Remove the PBS from the wells where the chips are placed and add 200 μL of PBS-Triton 0.5% for 30 min.
  b. Remove the PBS-Triton 0.5% and add 200 μL of blocking solution (10% donkey serum in PBS - Triton 0.5% and 2% BSA) for 1 h.
  c. Remove the blocking solution and add 200 μL of the primary antibody solution in PBS-Triton 0.5% and 2% BSA from 12 to 16 h.
  d. Wash with PBS-Triton 0.5% and 2% BSA for 5 min.
  e. Repeat step d 6 times.
  f. Prepare secondary antibodies solution in PBS-Triton 0.5% and 2% BSA. Add 200 μL to each well and incubate for 1 h.
  g. Wash with PBS for 5 min.
  h. Repeat step g 5 times.
  i. Remove the PBS and keep in 200 μL PBS – Pen/Strep 1%.

***Note:*** After staining, chip sections are kept in PBS-Pen/Strep 1% at 4 degrees to avoid any sample contamination. For imaging, the chips sections are placed on a slide with PBS and acquired as a regular section.

27. Chip 3D staining.
  a. Remove all the tips with PBS and replace with new tips in the gel channel and in the outlet of the endothelial channel.
  b. Add 100 μL of permeabilization solution (0.1% triton x-100 in PBS) and leave the tips in.
  c. Incubate the chips for 30 min at 20°C–25°C on a rocker.
  d. Remove all the tips and wash the channel with 200 μL PBS 3x for 15 min each.
  e. Remove all the tips with PBS and replace with new tips in the gel channel and in the outlet of the endothelial channel.
  f. Add a tip with 100 μL of blocking solution (5% BSA in PBS) to the bottom inlet and leave the tips in.
  g. Incubate the chips for 1 h at 20°C–25°C on a rocker.
  h. Remove all the tips and wash the channel with 200 μL PBS 3x for 15 min each.
  i. Prepare the primary antibodies solution for each channel in 1% BSA in PBS.
  j. Remove all the tips with PBS and replace with new tips in the gel channel and in the outlet of the endothelial channel.
  k. Add 100 μL of primary antibody solution to the bottom inlet and leave the tips in.
  l. Incubate the chips from 12 to 16 h at 4°C on a rocker.
  m. Remove all the tips and wash the channel with 200 μL PBS 3x for 15 min each.
  n. Prepare secondary antibodies solution in 1% BSA in PBS.
  o. Remove all the tips with PBS and replace with new tips in the gel channel and in the outlet of the endothelial channel.
  p. Add 100 μL of secondary antibody solution to the bottom inlet and leave the tip in.
    ***Note:*** From now on the chips should be covered and protected from light.
  q. Incubate the chips from 12 to 16 h at 4°C on a rocker.
  r. Remove all the tips and wash the channel with 200 μL PBS 3x for 15 min each.
  s. Remove all the tips with PBS and replace with new tips in the tumor channel and in the outlet of the endothelial channel.
  t. Add 100 μL of the nuclear staining solution (1:500 Hoechst in PBS) to the endothelial channel inlet and leave the tip in.
  u. Incubate for 4 h at 20°C–25°C in the dark, on a rocker.
  v. Remove all the tips and wash the channel with 200 μL PBS 3x for 15 min each.
    **Pause point:** Chips can be stored in the dark at 4°C with PBS in tips on all ports before imaging up to 2 weeks. Ensure that the chips never dry out during this time.
28. 3D Immunofluorescence imaging.
  ***Note:*** This protocol optimized imaging with the Leica Stellaris confocal microscope system. The following steps are proposed settings, and other platforms can be used.
  a. Place the stained chip on the microscope stage for imaging. Add PBS droplets on the channel ports to prevent premature dehydration during prolonged imaging sessions.
    ***Note:*** Ensure the chip is placed stable on the microscope table throughout image acquisition.
  b. Add the laser channels for each fluorophore used for staining.
    ***Note:*** When imaging multiple fluorophores, optimize acquisition parameters to minimize spectral overlap and channel crosstalk.
  c. Select an objective that provides sufficient working distance and field of view for volumetric imaging of the entire microfluidic channel.
    ***Note:*** Low-magnification objectives (e.g., 10×) are generally suitable for imaging large sample volumes along the Z-axis, whereas higher-magnification objectives may be used for detailed analysis of specific regions of interest.
  d. Define the imaging area and establish the upper and lower limits of the Z-stack to encompass the full thickness of the sample including top- and bottom channel.
  e. Acquire images at a minimum resolution of 1024 × 1024 pixels. Adjust scan speed, averaging, and detector settings to obtain an adequate signal-to-noise ratio while minimizing photobleaching.
  f. Optimize voxel sampling for three-dimensional reconstruction.
    ***Note:*** A Z-step size of ≤3 μm is recommended for routine volumetric imaging, while Z-step sizes of 1–2 μm provide improved image quality and more accurate 3D renderings. Smaller Z-step sizes increase acquisition time and data storage requirements.
    ***Note:*** The total acquisition time is influenced by the imaging volume, objective magnification, image resolution, number of fluorescence channels, tile number, averaging settings, and Z-step size. High-resolution, multi-channel volumetric datasets may require several hours to complete. For extended imaging sessions, acquisition strategies that prioritize sequential collection along the Z-axis may reduce imaging time and stage movement compared with repeated XY scanning.
  g. For large imaging volumes, acquire tiled image stacks when necessary to capture the entire channel area.
    ***Note:*** An overlap of 10% between adjacent tiles is recommended to facilitate accurate image stitching.
  h. Export image stacks in a format compatible with downstream image analysis software and generate three-dimensional renderings using suitable visualization software.
    ***Note:*** In this study, imaging was performed on a Leica Stellaris confocal microscope using a 10× dry objective, 1024 × 1024 acquisition format, a scan speed of 600 Hz, bidirectional X with Phase X on 17.14, and a Z-step size of 2.96 μm with Zoom Factor 4.38.
    ***Note:*** For representative immunofluorescence outcomes showing expected endothelial vessel formation in the bottom channel and tumor aggregation in the top channel, refer to Figure S3.

#### Cell retrieval

**Timing: 30 min**

This section describes the steps to follow for harvesting cells from both the gel and endothelial channels for further analysis such as flow cytometry or for gene/protein expression (Figure 3B).

***Note:*** Accutase will be used as a detachment solution for the ECs in the bottom channel and Corning Cell Recovery Solution will be used to recover the migrated PBMCs and tumor cells from the top channel.

**CRITICAL:** To ensure the viability of the cells after their retrieval from the chips, it is necessary to perform all the steps in a timely manner and not extend the recovery or detachment times; moreover, it is important to keep the cells/chips on ice when required.

29. Warm the Accutase at 37°C.
30. Label 2 Eppendorfs per chip, 1 for the gel channel and 1 for the endothelial channel.
31. After PBMC migration, rinse with 200 μL of PBS the inlet of the endothelial channel while aspirating the outflow to remove the non-adhered PBMCs.
32. Remove the PET disk from the inlet port of the gel channel.
33. Plug in empty tips in the outlet of the gel channel and in the inlet and outlet of the endothelial channel.
34. Add 200 μL of the cell recovery solution in the inlet of the gel channel and leave the tip in.
35. Pipette up and down without removing the tips to loosen/break up the gel.
36. Collect the volume of the gel channel in the gel channel Eppendorf.
37. Incubate on ice or at 4°C for 30 min.
38. Remove the empty tip from the inlet of the endothelial channel.
39. Add 100 μL of Accutase in the inlet of the endothelial channel and leave the tip in.
40. Place the chips in the incubator (37°C, 5% CO_2_) for 5 min.
41. Pipette up and down without removing the tips to loosen the cells.
42. Collect volume from the endothelial channel in the corresponding Eppendorf with 100 μL of EGM-2 MV (full) to inactivate the Accutase.
43. With another 100 μL of EGM-2 MV (full), rinse the channel of the chip and collect in the same Eppendorf.
44. Confirm proper cell detachment under the microscope.
45. If still many cells are observed to be attached to the channel under the microscope, repeat steps 42–52.
46. Spin down all the samples at 350 rcf for 5 min at 4°C.

***Note:*** As few cells are attached in the channels, both pellets will be very small and difficult to see.

47. Carefully remove the supernatant.
48. Resuspend in 50 μL of PBS.

### RNA/protein extraction and flow cytometry

**Timing: 30 min**

This section describes the steps to follow for further analysis such as flow cytometry or for gene/protein expression.

49. If for RNA/protein extraction:
  a. Spin down the selected samples at 300 rcf for 5 min at RT.
  b. Carefully remove the supernatant.
  c. Add 50 μL of cell lysis buffer and resuspend the cell pellet.
    **Pause point:** After cells are lysed, samples can be kept at −80°C for several months.
  d. If proceeding with the RNA or protein extraction, follow your regular protocol.
50. If for flow cytometry:
  a. Transfer the samples to a V-bottom 96 wells plate.
  b. Take 1 μL per sample to create your unstained and viability samples.
  c. Take half of your viability sample, freeze it shortly at −20°C to have your half-dead sample and pool it back in the plate.
  d. Spin down the plate at 350 rcf for 5 min at 4°C.
  e. Carefully remove the supernatant by inverting the plate.
  f. Resuspend the pellet in 50 μL blocking solution (Fc Receptor blocking in PBS – 1:200) for 10 min at RT.
  g. Prepare the antibodies solution (2x antibodies in MACS buffer – 0.5% BSA + 2 mM EDTA in PBS).
  h. Add 50 μL of the antibody solution.
    ***Note:*** Now on, the plate should be covered and protected from light.
  i. Incubate 30 min at 4°C in the dark.
  j. Add 75 μL of MACS buffer.
  k. Spin down the plate at 350 rcf for 5 min at 4°C.
  l. Carefully remove the supernatant by inverting the plate.
  m. Add 100 μL of 2% PFA.
  n. Incubate for 1 h at 20°C–25°C to fix the cells.
  o. Add 100 μL of MACS buffer and resuspend.
  p. Spin down at 350 rcf for 5 min at RT.
  q. Carefully remove the supernatant by inverting the plate.
  r. Add 200 μL of MACS buffer.
    **Pause point:** The plate can be stored for up to 3 days at 4°C, protected from light, before running the flow cytometry analysis.
  s. Measure samples on a suitable flow cytometer for the antibody panel detailed in key resources table.
    ***Note:*** The gating strategy used to identify and quantify immune cell subpopulations from the chip effluent is detailed in Figure S4.

### Secretome analysis

**Timing: 30 min**

This section describes the following steps to analyze the samples taken from the reservoirs and storage for further secretome analysis (Figure 3C).

51. If processing the samples collected after 24h of co-culture: (the media taken right before the perfusing with the PBMCs).
  ***Note:*** In this protocol, the supernatant samples were sent to NOMIC Bio (https://www.nomic.bio), where a multiplex ELISA was carried out.
  a. Spin down your samples at 300 rcf for 5 min to removes any debris or death cells present.
  b. Continue your analysis as described in the selected ELISA or multiplex.
    ***Note:*** If processing the samples collected after the PBMCs migration, the buoyancy media contains components that could interfere with the specific ELISA/multiplexing; extra steps might be needed to prepare the sample for analysis.

## Expected outcomes

The above protocol describes how to establish a TME-on-a-chip that recapitulates immune-hot and immune-cold contexts using human LUAD cell lines, primary HMVECs-L, and human PBMCs. After completion of the protocol, immune cell migration can be analyzed, migrated immune-cell populations can be characterized by flow cytometry, and the cytokine secretome of the co-culture system can be assessed.

After the three-day vessel formation protocol, primary HMVECs-L are expected to form a confluent vascular bed in the bottom channel, as observed by bright-field microscopy. Tumor cell lines seeded in the top channel (H2009 cells for immune-hot, A549 cells for immune-cold) should adhere and proliferate within the matrix, resulting in a dense cellular layer visible at both 4x and 10x magnification. The chip assembly typically requires 4 days from activation to PBMC recruitment. Endothelial confluence and tumor cell attachment should be verified by bright-field imaging prior to the immune recruitment step.

After 4 h of PBMC recruitment, immune cells are expected to transmigrate from the endothelial channel toward the tumor channel. 3D confocal rendering of the whole chip should reveal CD45^+^ immune cells distributed between the endothelium and tumor compartments. Cross-sectional immunofluorescence imaging can be used to confirm the spatial organization of the two compartments, with tumor cells and ECs (VE-cadherin^+^) distinguishable across the chip layers.

For flow cytometry analysis, cells are expected to be retrieved from both the upper (tumor) and bottom (vascular) channels. Using the gating strategy described (FSC/SSC gating, singlet discrimination, live/dead exclusion with efluor 780, and CD45/CD31 separation), researchers should expect significantly more CD45+ immune cells to migrate into the upper channel of immune-hot (H2009) chips compared to immune-cold (A549) chips. Among the immune subpopulations, CD8+ T cells and NK cells (CD56+) show significantly increased migration toward the immune-hot tumor, while total T cells (CD3+), CD4+ T cells, and B cells (CD19+) trend higher but may not reach statistical significance.

Secretome analysis of the effluent collected from the outlet bottom channel after 24h of co-culture should reveal distinct secretory profiles between the two tumor contexts. Pathway enrichment analysis (Enrichr, MSigDB Hallmark 2020) of cytokines upregulated in the immune-hot condition is expected to identify pro-inflammatory pathways. The secretome data demonstrate that the chip generates a biologically relevant cytokine milieu that distinguishes immune-hot from immune-cold TME.

Following chip establishment, isolated cells and conditioned media can be used for additional downstream assays not addressed in this protocol, including bulk or single-cell RNA sequencing of cancer cells, ECs, and migrated immune populations, or targeted ELISA validation of candidate cytokines.

## Limitations

### Cell lines

This protocol was optimized using two lung cancer tumor cell lines (A549 and H2009) and HMVEC-L for vessel formation. When using other cell lines, users should adjust key parameters, including cell seeding density, culture conditions, and the timing required to achieve a fully confluent vessel channel, to ensure reproducibility in other cellular contexts.

### Limited microenvironment complexity

This model represents a 3D TME comprising three principal components: tumor, immune, and ECs. In the current context, this allows for the focused study of the immune microenvironment including immune cell interaction and infiltration across the endothelium. However, it does not include fibroblasts, which are important contributors to tumor-stroma dynamics, or pericytes, constituents of the perivascular niche, and contributors to vascular stability and function. The system could be further expanded by incorporating other components of the TME to better recapitulate its complexity.

### Limited material

Immune cell transmigration into the tumor channel is limited, as the number of cells that are able to transmigrate under the flow for 4 h is reduced, which constrains flow cytometry analysis of specific subpopulations, especially lower-frequency immune subsets and populations requiring intracellular staining, the latter being further affected by cell loss during staining and washing procedures. Extending the perfusion period and the number of perfusions could potentially increase the number of infiltrated cells, although this has not been tested. Similarly, the low number of endothelial and tumor cells per chip yields limited RNA, DNA, and protein, often requiring pooling multiple chips or the use of low-input kits for downstream assays.

### Chip platform specificity

This protocol was optimized for S1 chips. Other chip designs, such as Emulate’s R1 chips, may require optimization of cell seeding densities, culture times, and other experimental parameters to achieve comparable vessel formation and immune cell infiltration.

### Cytokine analysis limitations

Cytokine analysis is limited once immune cells are added in buoyancy medium, as the medium itself generates background signal, making it difficult to accurately quantify secreted cytokines. This restricts downstream immunoassays unless alternative collection strategies or media are employed.

### Culture period constraints

Tumor cells are seeded in Matrigel 24 h prior to the immune cell flow. Due to their high proliferation rate, tumor cells degrade the Matrigel over time, limiting the duration of co-culture experiments. Parameters such as tumor cell number, Matrigel concentration, or co-culture timing may need to be adjusted to maintain matrix integrity and ensure experimental stability.

## Troubleshooting

### Problem 1

At all stages of the experiment, Emulate chips must be inspected for air bubbles before, during, and after introducing new solutions or cells, as bubble formation may disrupt cell attachment and viability, and could compromise stable flow.

### Potential solution

• Air bubble formed during introduction of any solution in the absence of cells (step 1):

Fully aspirate the solution from the affected channel and slowly reintroduce the appropriate fresh solution (coating solution, medium, or PBS). Ensure that the channels do not dry out during solution exchange by introducing the new solution swiftly.

• Air bubbles formed after ECs have been seeded in the bottom channel (step 3): Prepare a new 200 μL pipette tip and load it with 180 μL of medium, leaving an air pocket at the bottom of the tip. Connect the pipette tip to the inlet of the bottom channel and slowly introduce the air into the channel, followed by the medium. The initial large air bubble will help displace smaller trapped bubbles, after which fresh medium will enter the channel. Introduce the air and medium carefully and gradually to minimize mechanical stress and reduce the risk of EC detachment.
• Air bubbles are detected in the Matrigel of the top channel (step 5): Air bubbles trapped in the Matrigel can only be removed immediately before the gel is fully polymerized. If a bubble is observed, promptly aspirate the cell-gel mixture from the top channel and immediately refill the channel with medium to prevent drying. Prepare a fresh batch of gel-cell suspension and repeat the seeding procedure. To accommodate potential reseeding, it is therefore recommended to prepare sufficient cells and reagents in advance. During preparation of the gel mixture, avoid introducing air while resuspending the gel to minimize bubble formation. If microscopic air bubbles persist in the top channel, the Regulate Cycle from Emulate could potentially remove these bubbles, while also increasing the risk of unstable gel.

### Problem 2

During preparation of the gel-cell mixture (step 5), the gel may prematurely polymerize before it is introduced into the top channel. Premature polymerization can result in a thin, unstable gel that is unable to support both tumor cells and PBMCs effectively.

### Potential solution

• Premature crosslinking of gel and thin gel formation: Prepare the gel-cell mixture on ice using cold reagents and pre-chilled sterile pipette tips. If premature polymerization occurs, a thin gel structure can be observed under the microscope immediately after seeding. In this case, promptly remove the mixture and prepare a fresh solution. It is important to work efficiently during preparation to minimize gel crosslinking at room temperature (20°C–25°C) and ensure consistent gel structure.

### Problem 3

Under flow conditions (steps 11–14, 22–26), the gel in the top channel may detach from the channel walls if the gel-cell mix was prepared incorrectly. Gel detachment can lead to loss of cells and compromise the detectability of PBMCs in the top channel.

### Potential solution

• Unstable gel: Stable gel adhesion is supported by proper ECM coating of the top channel. Ensure that the ECM solution is introduced without persisting air bubbles, as this coating helps maintain gel connection to the channel walls. Similarly, introduce the gel itself carefully, to avoid air bubbles during loading. When connecting the chip to the pod (step 8–9), verify that the PET disks are correctly positioned. Even small gaps between the vias and the PET disk can allow air entry and potentially destabilize the gel. It is also important not to increase the concentration of tumor cells in the gel beyond what is specified in the protocol, as higher cell densities can negatively affect gel structure. Any modifications to cell concentrations or handling procedures should be tested and validated experimentally to ensure stability.

### Problem 4

Before initiating flow, ensure that the PET disks are correctly positioned on the top channel vias and inspect the channels for air bubbles (during step 5). Improper positioning or trapped air can disrupt flow and compromise experimental outcomes.

### Potential solution

• Solutions degasification: Both the culture medium and the buoyancy medium must be properly degassed before using in the chips during all flow conditions (steps 6, 16). Trapped air in the medium can lead to bubble formation, obstruct channels, and compromise expected outcomes. This also accounts if priming the pods was done insufficiently.
• Clog during the introduction of PBMCs into the chip: If the chip becomes clogged during the introduction of PBMC in buoyancy medium into the bottom channel (steps 25–27), this may indicate that the buoyancy medium was prepared incorrectly. Alternatively, insufficient resuspension of PBMCs can cause cells to aggregate, leading to channel blockage. Therefore, gently resuspend PBMCs before introduction, verify correct medium preparation and viscosity, and confirm that all channels are free of bubbles and debris prior to introducing flow. Maintaining these precautions ensures consistent flow and minimizes experimental variability.
• Cells viability: It is also important to check the viability of the seeded cells, as a high number of detached or dead cells can accumulate and obstruct the pod channels. If clogging occurs during flow conditions, stop the Zoë machine and carefully disconnect the chip from the pod. Using a pipette, slowly and gently introduce an appropriate medium into the bottom channel to clear the obstruction. Avoid applying excessive pressure, as this may dislodge adherent cells in the bottom channel or disrupt the gel structure in the top channel through pressure through the pores. After unclogging, inspect the channel to ensure that flow can be restored and no air bubbles remain.

### Problem 5

After completing PBMC transmigration, cells can be harvested from the chip for downstream analysis, such as flow cytometry or qPCR (steps 58, 59). Efficient digestion of the gel and thorough accutase treatment are critical to recover enough cells.

### Potential solution

• Cell retrieval problems: To maximize cell retrieval, introduce the cell retrieval solution throughout the entire top channel using gentle mechanical agitation. Move the solution back and forth to fully resuspend and digest the gel, ensuring that cells are released into suspension. For EC harvest from the bottom channel, an additional preparatory step can improve recovery. First, collect the medium from the bottom channel and wash the channel with PBS, collecting this wash as well (before step 48). After these initial steps, introduce the accutase solution to detach adherent ECs efficiently (step 48). Careful handling during these steps minimizes cell loss and ensures maximal recovery for downstream applications.

### Problem 6

Differences in refractive index between the PBS in the bottom channel and the Matrigel® in the top channel can complicate 3D imaging. Instruments such as the Leica Stellaris 5 and 8 are capable of correcting these refractive index mismatches. However, high background fluorescence or low target signal may still occur.

### Potential solution

• Low signal during immunofluorescence imaging: To minimize background and improve signal detection, ensure that all washing steps are performed for the full duration recommended in the protocol (step 36). Adequate washing reduces nonspecific antibody binding and lowers background fluorescence. Use a rocker to enhance distribution of antibodies and washing solutions throughout the channels, promoting uniform staining (step 36). If a fluorophore signal during imaging was lower than expected, a re-staining by repeating the staining protocol can be done (starting at step 36 h). Post-acquisition image processing, including deconvolution and 3D rendering, can further improve signal-to-noise ratio and clarity of reconstructed images.

In addition, antibodies used in this protocol were selected for compatibility to avoid spectral overlap. Be aware that combining multiple fluorophores, or detecting low-abundance proteins, can lead to photobleaching or signal masking, which reduces the detectability of certain targets. If users deviate from the fluorophore combinations specified in this protocol, optimization of fluorophore selection and sequential staining may be required to ensure accurate imaging.

### Problem 7

The appropriate section thickness depends on the experimental goal: thin sections improve signal resolution for imaging, while thicker sections capture greater cellular diversity. The cells and gel are sensitive to collapse during the cutting procedure, which may lead to loss of cells.

### Potential solution

• Sectioning problems: Before sectioning, carefully inspect the gel integrity in the top channel (before step 33). Only chips with gels that maintain structural stability should be selected for sectioning, as collapsed or unstable gels are poor candidates for thinner sections. Maintain hydration in all channels during preparation to prevent drying, since dried cells or gel may detach during the cutting procedure and compromise the sections (step 33). Sectioning should be performed promptly, ideally within two weeks after completing PBMC transmigration. Over time, the gel composition may degrade due to gravity and natural decomposition, reducing structural integrity and experimental reliability. To ensure sufficient material for downstream analyses, cut multiple sections from each chip.

In all cases, chips with good structural and cellular integrity will yield complete and stable sections suitable for analysis.

## Resource availability

### Lead contact

Further information and requests for resources and reagents should be directed to and will be fulfilled by the lead contact, Jeffrey Kroon (j.kroon@amsterdamumc.nl).

### Technical contact

Technical questions on executing this protocol should be directed to and will be answered by the technical contact, Patricia Yagüe (patricia.yagueesanz@kuleuven.be).

### Materials availability

Data generated by this study are available from the lead contact upon request.

### Data and code availability

This study does not report original code.

## Acknowledgments

P.C. is supported by grants from Methusalem funding (Flemish government, METH/21/06), funded by the European Union: European Research Council (ERC) Advanced Research Grant (101055155), ERC-2024-Proof of Concept grant (101213917), the Fund for Scientific Research-Flanders (FWO-Vlaanderen, G088121N, G0B7920N), and the Khalifa University & VIB-KU Leuven Biomedical Science Discovery Program (BISDI). S.B.A. is supported by Methusalem funding (Flemish government, METH/21/06). A.R.C. is supported by Inserm and received grants from the French National Cancer Institute (PRT-K 2023-113 and INCa 20174). P.Y. is supported by HORIZON-MSCA-2023-Postdoctoral fellowship (101154254 – noCOLDmore). J.K. is supported by the Dutch Heart Foundation (Senior Scientist Dekker grant 03-004-2021-T045) and an NWO-Vidi grant (09150172310053), and S.v.K. and J.K. are funded by the European Union (ERC, ENDOMET-STEER, 101076407). Views and opinions expressed are, however, those of the authors only and do not necessarily reflect those of the European Union or the European Research Council Executive Agency. We would like to acknowledge the technical support of Dr. S. Vinckier and A. Manderveld (VIB – KU Leuven) for staining optimization, Katie Hanford (Amsterdam UMC) for her guidance, as well as the VIB Flow Core Facility at VIB Leuven for their guidance with panel design in this study. Finally, we would like to thank the volunteers who donated blood for this study. Graphical abstract and figures were created using Biorender.com.

## Author contributions

S.B.A. and P.Y. conceptualized and developed the model, performed the experiments, analyzed the data and results, and wrote and revised the manuscript. S.v.K. performed the experiments, analyzed the data and results, and wrote and reviewed the manuscript. A.R.C. provided constructive feedback on the work. J.K. contributed to the conceptualization and development of the model, reviewed the manuscript, and provided funding support. P.C. reviewed the manuscript and provided funding support.

## Declaration of interests

The authors declare no competing interests.
