# Supplementary material for: Protocol for generation of a 3D humanized lung-tumor-on-a-chip model of immune-hot and immune-cold microenvironments
Source: STAR Protoc. 2026 Jul 23;7(3):104732. doi: 10.1016/j.xpro.2026.104732 (PMC13425899; doi:10.1016/j.xpro.2026.104732)
Supplement: Document S1. Figures S1–S4 [file mmc1.pdf]

Figure S1. Cell seeding, related to Step 4. (**A**) Representative bright-field images of the middle bottom channel during the three-day vessel formation process. (**B**) Representative bright-field images of the upper channel taken after 24h seeding. Scale bar: 200 µm.

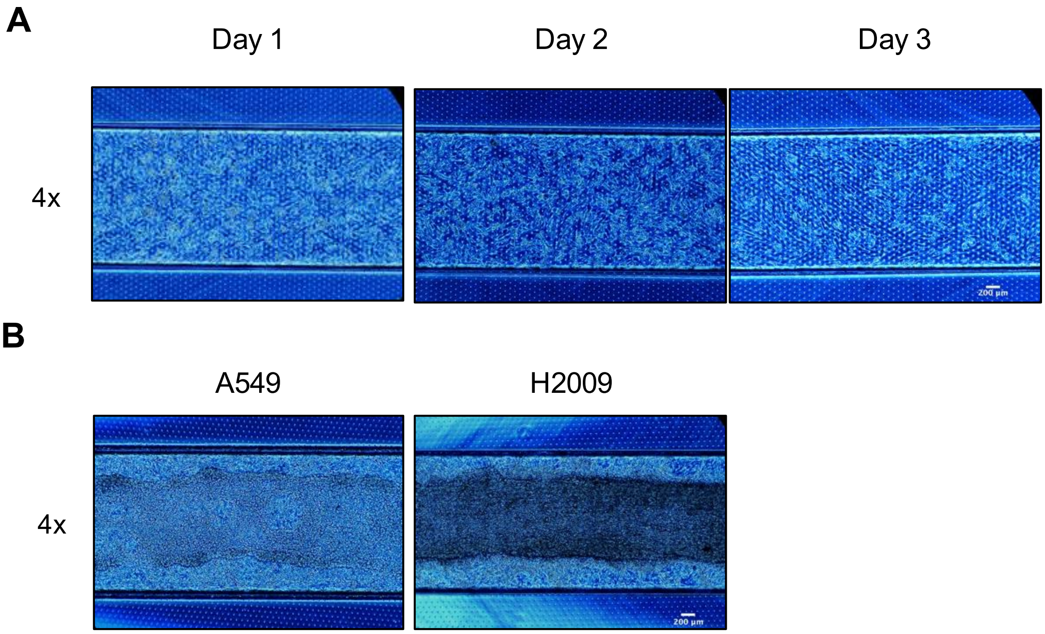

Figure S2. Closed channel chip with tips, related to Step 24. b

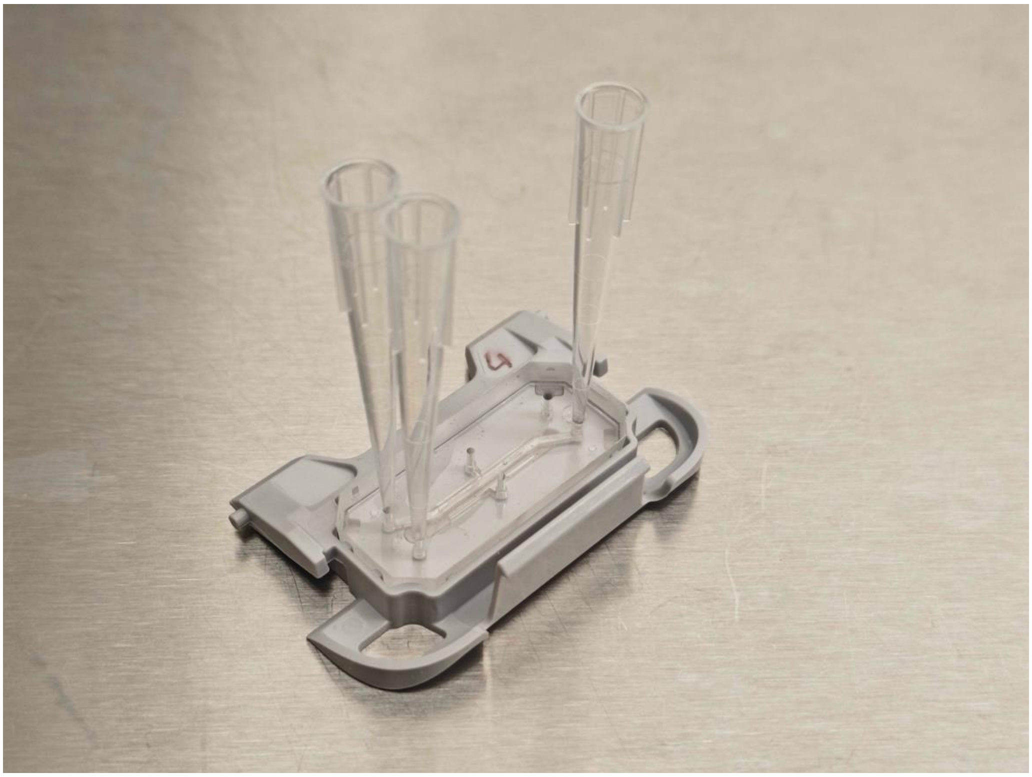

Figure S3. Representative 3D confocal reconstructions of the tumor-microenvironment-on-chip, related to Step 28. (A) Top-view renderings of the bottom endothelial channel (left) and the top tumor channel (right), showing endothelial vessel formation and tumor cell aggregation, respectively. (B) Side-view (orthogonal) reconstructions highlighting the vessel-like structure formed by endothelial cells in the bottom channel and the spatial organization of tumor aggregates in the upper channel. Tumor cells were labeled with CellTracker (red), endothelial cells with VE-cadherin (orange), and nuclei with Hoechst (blue). Scale bar: 100 μm.

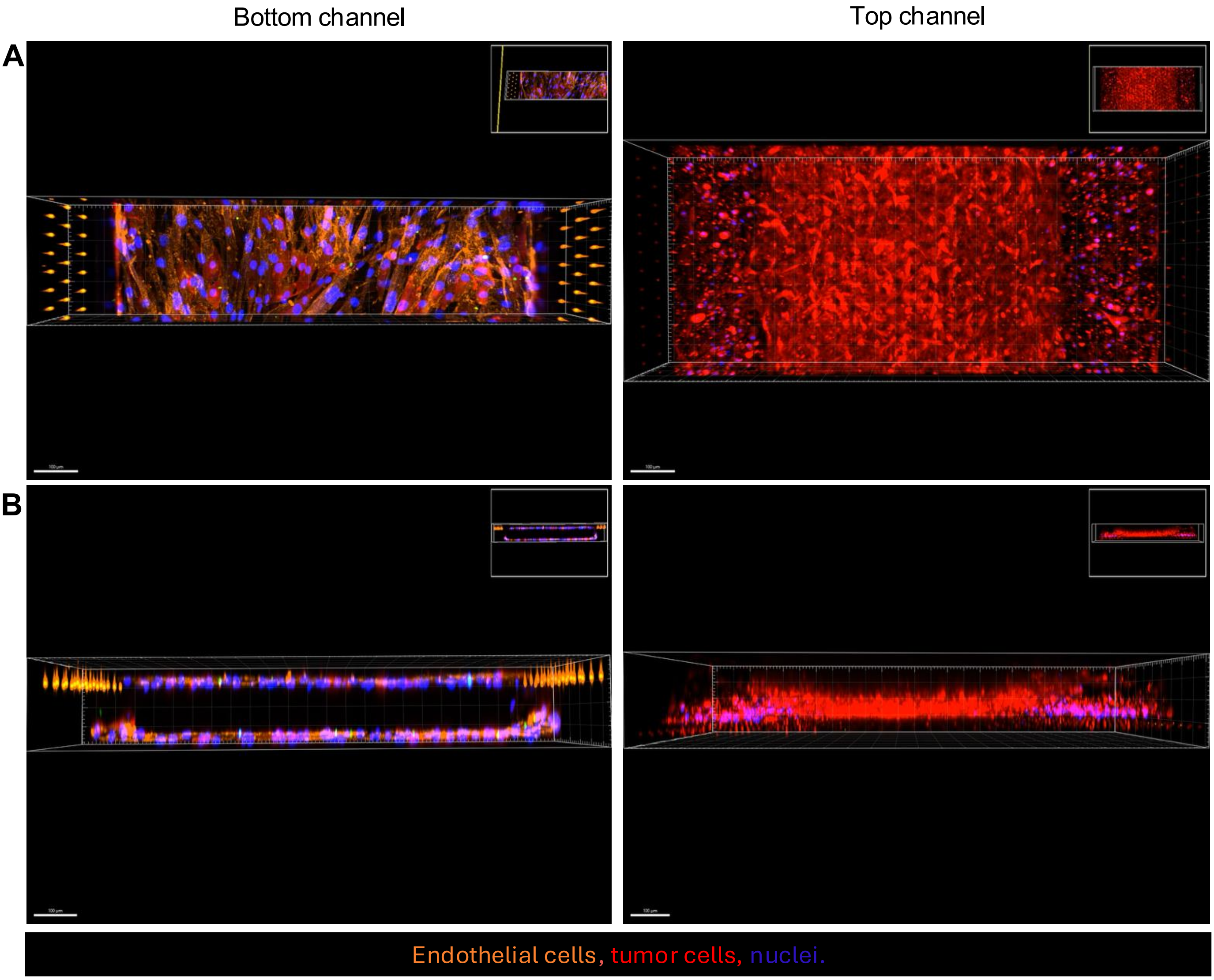

Figure S4. Gating strategy, related to Step 50, of: (A) immune cells and ECs retrieved from channel by flow cytometry. Cells were gated from forward and side scatter area (FSC-A and SSC-A respectively), singlets were gated based on the FSC-A and forward scatter height (FSC-H). Live immune cells were gated from CD45+, efluor 780- and live endothelial cells (CD31+, efluor 780-); **(B) migrated PBMCs retrieved from tumor channel (upper channel) by flow cytometry.** Cells were gated from forward and side scatter area (FSC-A and SSC-A respectively), singlets were gated based on the FSC-A and forward scatter height (FSC-H). Live immune cells were gated from CD45+, efluor 780-. CD3 was used to identify T cells (CD3 +) or non-T cells (CD3 -). From CD3 + T cells, CD4 and CD8 T cells were gated using CD4 and CD8 antibodies. From CD3 -T cells, staining with CD19 antibody for B cells.

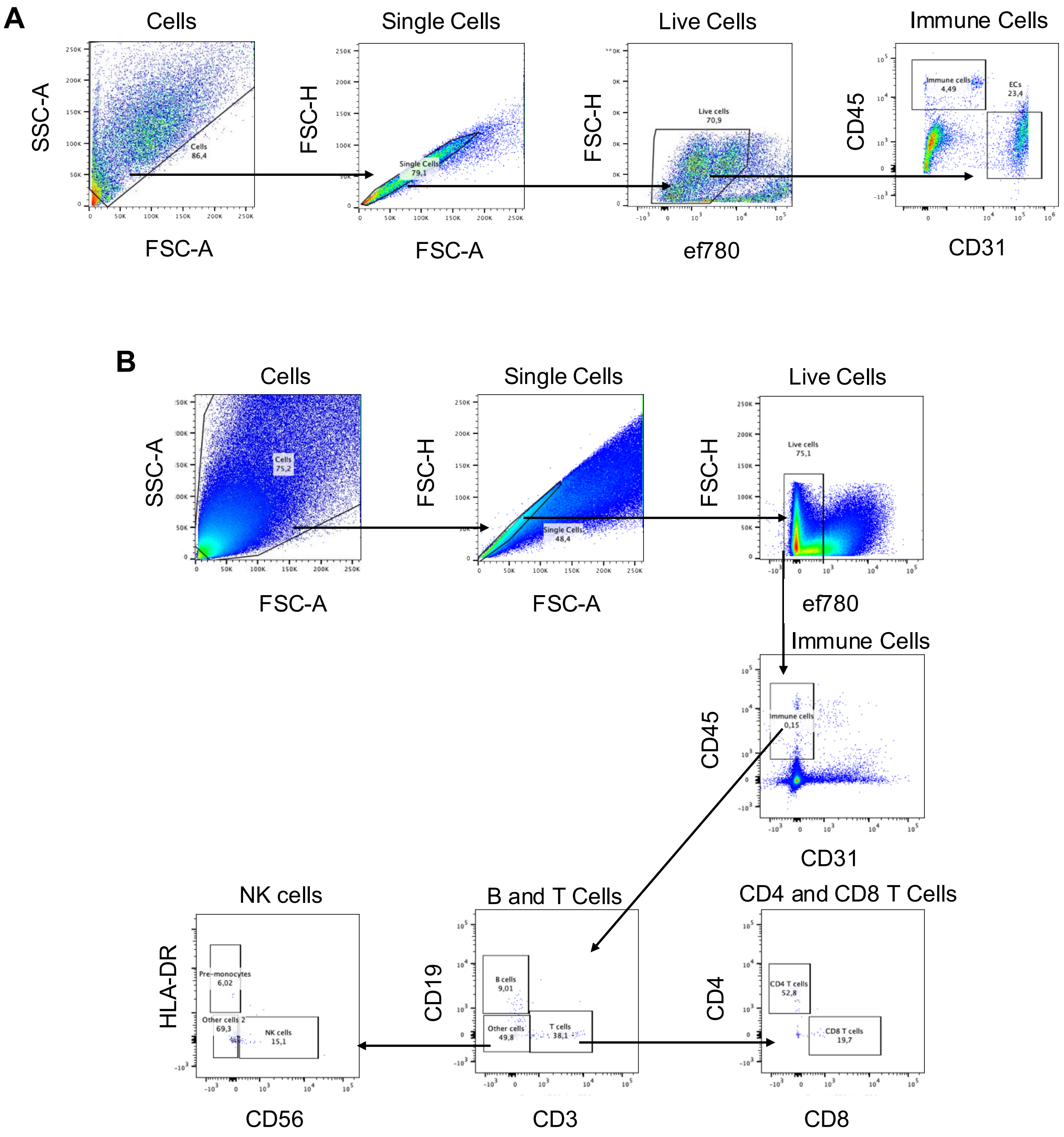

Video S1. Step-by-step procedure for chip sectioning, related to Step 25.
